# Supplementary material for: Japanese clinical practice patterns of rituximab treatment for minimal change disease in adults 2021: A web-based questionnaire survey of certified nephrologists
Source: PLoS One. 2024 Mar 29;19(3):e0299053. doi: 10.1371/journal.pone.0299053 (PMC10980199; doi:10.1371/journal.pone.0299053)
Supplement: S1 Text — (DOCX) [file pone.0299053.s001.docx]

**S1 Text**

**Questionnaire Items**

**1. Rituximab (RTX) for minimal change disease (MCD) in adults**

*Q1. Do you administer RTX for adult MCD in your current facility? If you do, please choose one option close to the number of cases that you administer RTX for.*

- Neither administer nor refer to other facilities

- Do not administer but refer to other facilities

- Less than 1 case per year on average

- 1 to 5 cases per year on average

- 6 to 20 cases per year on average

- More than 20 cases per year on average

*Q2. This question is for those who answered yes in Q1. Which is the funding source of RTX for adult MCD cases (multiple choices allowed)?*

- Patient payment (covered by insurance)

- Patient payment (not covered by insurance)

- Hospital payment (not covered by insurance)

- Research funding from the clinical department

- Research funding from individual physicians

- Unknown

- Others

**2. Withholding of RTX administration for MCD in adults**

*Q3. Have you ever withheld (or will you withhold hereafter) RTX administration for adult MCD (multiple choices allowed)? If you have withheld or will withhold, please provide your reason, excluding the effects of the COVID-19 pandemic and patient factors such as allergic reactions to RTX and coexisting malignancies.*

- No withholding

- Yes (Because the cost is not covered by insurance, and a funding source is difficult to secure)

- Yes (Because the cost is not covered by insurance, and the facility or the ethical committee prohibits its usage)

- Yes (Because of limited experience in its usage)

- Yes (Because of an inadequate medical care system for possible complications of RTX treatment in the facility)

- Impossible to answer because I have never been in charge of a case like this

- Others

**3. RTX regimens for frequently relapsing nephrotic syndrome in adults**

*Q4. Please indicate the dosage of RTX in the introduction period.*

- Impossible to answer because of my limited experience in its usage

- Lower than a body surface area (BSA)-based dose of 375 mg/m^2^

- BSA-based dose of 375 mg/m^2^

- More than a BSA-based dose of 375 mg/m^2^

- Others

*Q5. Please indicate the administration protocol of RTX in the introduction period.*

- Single-dose administration

- Twice at 1-week intervals

- Four times at 1-week intervals

- Twice at 2-week intervals

- Others

*Q6. Please indicate the interval of RTX administration in the maintenance period with sustained remission.*

- No administration in the maintenance period with sustained remission

- 6 months

- 7 to 11 months

- More than 12 months

- Others

*Q7. Please indicate the duration of RTX administration in the maintenance period with sustained remission.*

- Less than 1 year

- 1 to 2 years

- 2 to 3 years

- 3 to 5 years

- More than 5 years

- Others
