# Supplementary material for: Japanese clinical practice patterns of rituximab treatment for minimal change disease in adults 2021: A web-based questionnaire survey of certified nephrologists
Source: PLoS One. 2024 Mar 29;19(3):e0299053. doi: 10.1371/journal.pone.0299053 (PMC10980199; doi:10.1371/journal.pone.0299053)
Supplement: Attachment — Submitted filename: R1_ResponseToReviewers_231202.docx [file pone.0299053.s002.docx]

**Detailed Response to Reviewers**

**Title: Japanese Clinical Practice Patterns of Rituximab Treatment for Minimal Change Disease in Adults 2021: A Web-Based Questionnaire Survey of Certified Nephrologists**

We sincerely appreciate all the Reviewers’ valuable comments. They helped us improve our manuscript, and we have taken them into account while revising our manuscript, as described below. Our revisions in the manuscript made based on Reviewer 1's comments are highlighted in green, and Reviewer 2's, in yellow.

**Reviewers’ comments:**

**Reviewer #1**

*This manuscript described the Japanese clinical practice patterns of rituximab treatment for minimal change disease in adults from nation-wide**, cross-sectional questionnaire survey. Very important issue, but there are several concerns that should be addressed.*

1. *Table 3: Please specify the confounding factors that were entered into the multivariate analysis; was location of affiliation not included?*

**Response:** We apologize for our insufficient description. In this multivariate analysis, a generalized estimating equation modeling method was adopted, so the working facility was chosen as the cluster unit rather than as a covariate. By using the working facility as the cluster unit, we were able to correct the magnitude of the contribution to the estimates produced by the responses of a large number of nephrologists within the same facility (Hanley JA et al. Statistical analysis of correlated data using generalized estimating equations: an orientation. Am J Epidemiol. 2003;157:364–375). To explain this, we have added this to the table footnote as follows (Table 3):

“A generalized estimating equation was fit to estimate the proportionate difference in the experience while considering the clustering of nephrologists in the same facility with the independent variables of years of experience as a physician, type of affiliation, number of kidney biopsies performed at the affiliation, number of patients with primary nephrotic syndrome treated in an outpatient setting, and the population density of the location of the affiliation.”

We did not rigorously assume confounding factors because this multivariate analysis was exploratory. Therefore, please note that the variables used to estimate the association are referred to as independent variables.

On the other hand, in the statistical analysis portion of the Methods section, there was a lack of description of the explanatory variables, so we have amended the relevant part as follows:

“To explore factors associated with the experience of RTX use at the current affiliation, a generalized estimating equation with robust variance estimation (with an identity link function and a Gaussian distribution family) was fitted with the following independent variables: years of experience as a physician, type of affiliation, **number of kidney biopsies performed at the affiliation,** number of patients with primary nephrotic syndrome treated in an outpatient setting, and population density of the location of the affiliation [9].”

2. *The survey results are shown for each individual, but are the results the same for each facility?*

**Response:** Unfortunately, we were unable to survey facility-specific policies regarding RTX use. Therefore, we have added to the limitations portion of the Discussion section as follows:

“Finally, we were not able to examine the regulations regarding RTX use on a facility basis. However, we believe that physician-level summary data would also be useful, for example, the funding sources of RTX treatment in Table 4, because discretionary authority may vary by job position even among nephrologists at the same facility.”

3. *Why is "Patient payment (covered by insurance)" done even though it is not provided by insurance coverage?*

**Response:** We apologize for the insufficient explanation. RTX is approved in Japan only for children with FR/SD MCD. However, our data can also be interpreted to reveal the reality that reimbursement is approved in adult cases without being deemed inappropriate by the authorities. To clarify this, we have added the following sentence to the Discussion section:

“On the other hand, this study also revealed that the majority of RTX use in adult FR/SD MCD cases was approved for reimbursement without being assessed as inappropriate by the authorities.”

**Reviewer #2**

*This article investigated the clinical practice patterns of rituximab treatment for minimal change disease (MCD) in adults in Japan. The authors conducted a web-based survey of certified nephrologists and analyzed the factors associated with the use of rituximab, the barriers to its use, and the variation in the treatment regimens for adult MCD. It addresses a relevant and timely topic, as rituximab is a promising therapy for frequently relapsing or steroid-dependent MCD, but its efficacy, safety, and cost-effectiveness in adults are not well established, and its reimbursement by insurance is not explicit in Japan.*

1. *Weaknesses of this article also include: It relies on self-reported data, which may be subject to recall bias, social desirability bias, or inaccurate reporting by the respondents.*

**Response:** In accordance with the reviewers' comments, we have added a statement to the limitations portion of the Discussion section regarding the information bias associated with self-reporting:

“Third, there may have been information bias due to self-reporting on the percentages of RTX use, such as recall bias and social desirability bias. To determine a more accurate actual status of RTX use, a large database study linking patient data from medical records with physician data is necessary.”

2. *It does not compare the outcomes or effectiveness of different rituximab regimens or dosages, which limits the ability to draw conclusions about the optimal treatment strategy for adult MCD.*

**Response:** We agree with the reviewer. We have added the following sentence to the Discussion section to address the need for effectiveness studies based on patient data:

“For the identification of the optimal treatment strategy for adult FR/SD MCD, further studies with patient data to analyze the effectiveness of those RTX regimens and doses are warranted.”

3. *It does not explore the patients’ perspectives, preferences, or experiences with rituximab treatment, which may affect the adherence, satisfaction, and quality of life of the patients.*

**Response:** We agree with the reviewer. If patient-reported outcomes could be added to patient-level data studies, differences in treatment adherence, satisfaction, and quality of life would also become clear. We have added this to the Discussion section as follows:

“If patient-reported outcomes could be added to such studies, differences in treatment adherence, satisfaction, and quality of life would also be revealed.”

4. *It does not discuss the limitations, implications, or recommendations of the study, which may reduce the impact and applicability of the findings.*

**Response:** As we have responded above, we have added limitations and future issues to the Discussion section.
