# Supplementary material for: Japanese clinical practice patterns of rituximab treatment for minimal change disease in adults 2021: A web-based questionnaire survey of certified nephrologists
Source: PLoS One. 2024 Mar 29;19(3):e0299053. doi: 10.1371/journal.pone.0299053 (PMC10980199; doi:10.1371/journal.pone.0299053)
Supplement: Attachment — Submitted filename: R2_ResponseToReviewers_231223.docx [file pone.0299053.s003.docx]

**Detailed Response to Reviewers**

**Title: Japanese Clinical Practice Patterns of Rituximab Treatment for Minimal Change Disease in Adults 2021: A Web-Based Questionnaire Survey of Certified Nephrologists**

We sincerely appreciate all the editor’s valuable comments. They helped us improve our manuscript, and we have taken them into account while revising our manuscript, as described below. Our revisions in the manuscript made based on the editor's comments are highlighted in yellow.

**Academic editor’s comments:**

*The manuscript is of interest to the nephrology community, but requires more detailed methodology description.*

1. *How many nephology in-patient and out-patient centers are there in Japan?*

**Response:** The number of clinics (defined as medical facilities having ≤20 inpatient beds) and hospitals offering nephrology as a medical specialty in 2020 is 2154 and 1381, respectively [[1]](https://paperpile.com/c/g740IO/cI0hS). We have added this to the Participants portion of the Methods section.

Reference

1.	[Ministry of Health, Labour and Welfare. Summary of Static/Dynamic Survey of Medical Institutions and Hospital Report, 2020 (in Japanese). In: Ministry of Health, Labour and Welfare [Internet]. 27 Apr 2022 [cited 19 Dec 2023]. Available:](http://paperpile.com/b/g740IO/cI0hS) <https://www.mhlw.go.jp/toukei/saikin/hw/iryosd/20/dl/09gaikyo02.pdf>

2. *How many of these centers perform kidney biopsies?*

**Response:** As of July 18, 2023, there are 715 teaching facilities accredited by the JSN [[1]](https://paperpile.com/c/g740IO/AoVVH). Although an exact number of accredited facilities performing kidney biopsies is unavailable, the 2018 survey on nationwide kidney biopsy practices targeted these accredited facilities [[2]](https://paperpile.com/c/g740IO/5vzSM). We have added this to the Participants portion of the Methods section as follows:

“As of July 18, 2023, there are 715 teaching facilities accredited by the JSN [[1]](https://paperpile.com/c/g740IO/AoVVH). Although the exact number of facilities performing kidney biopsies is unavailable, we believe that the number is close to the number of these accredited teaching facilities.”

References

1.	[Japanese Society of Nephrology. Teaching facilities accredited by the Japanese Society of Nephrology (715 facilities as of July 18, 2023) (In Japanese). In: Japanese Society of Nephrology [Internet]. 18 Jul 2023 [cited 19 Dec 2023]. Available:](http://paperpile.com/b/g740IO/AoVVH) <https://jsn.or.jp/jsninfo/about/facilities/>

2.	[Kawaguchi T, Nagasawa T, Tsuruya K, Miura K, Katsuno T, Morikawa T, et al. A nationwide survey on clinical practice patterns and bleeding complications of percutaneous native kidney biopsy in Japan. Clin Exp Nephrol. 2020;24: 389–401. doi:](http://paperpile.com/b/g740IO/5vzSM)[10.1007/s10157-020-01869-w](http://dx.doi.org/10.1007/s10157-020-01869-w)

3. *How many nephrologists certified by the Japanese Society of Nephrology (JSN) were practicing in Japan at the time of the survey? How may were invited to participate in the survey? How many responded to the invitation?*

**Response:** There were 5,777 certified nephrologists at the time of the survey [[1]](https://paperpile.com/c/g740IO/tlnYj). The sampling method used was convenience sampling, using the mailing list for JSN members or direct mailing by members of the working group to nephrologists of their acquaintance [[2]](https://paperpile.com/c/g740IO/FKBeU). We have added this to the Participants portion of the Methods section.

Because we invited respondents via convenience sampling for the survey, the total number of individuals who were invited was unfortunately unavailable. Thus, we have added the following sentence to the limitation portion of the discussion section:

“In addition, the total number of individuals who were invited to complete the survey was unavailable.”

The number of respondents was 434, as documented in the Results section.

References

1.	[Japanese Medical Specialty Board. List of the numbers of members and certified physicians of each society (as of August 2021) (In Japanese). In: Overview of the Japanese medical specialty system, 2021 [Internet]. Mar 2022 [cited 19 Dec 2023]. Available:](http://paperpile.com/b/g740IO/tlnYj) <https://jmsb.or.jp/wp-content/uploads/2022/04/gaiho_2021.pdf>

2.	[Wada T, Shimizu S, Koizumi M, Sofue T, Nishiwaki H, Sasaki S, et al. Japanese clinical practice patterns of primary nephrotic syndrome 2021: a web-based questionnaire survey of certified nephrologists. Clin Exp Nephrol. 2023. doi:](http://paperpile.com/b/g740IO/FKBeU)[10.1007/s10157-023-02366-6](http://dx.doi.org/10.1007/s10157-023-02366-6)

4. *Please provide a map of the centers, indicating the number of practicing nephrologists and the number of nephrologists who completed the survey.*

**Response:** We have provided a Google My Map representing the working facilities of the nephrologists who responded to the survey and have named it Figure 1 ( <http://tinyurl.com/4j2ct6s9> ). The number of nephrologists per facility who completed the survey was one in 77% (215 facilities) of the facilities, two in 13% (37 facilities), and three or more in 9% (26 facilities). Unfortunately, the number of nephrologists working at the facility was not available. We have added the following sentence to the Results section:

“The locations of which the ZIP codes (assuming there was only one nephrology provider in that district) were correctly identified among the 278 facilities are shown in Figure 1.”

As responded in comment #3, 434 nephrologists responded to the survey.
